# Characterization of Silver Nanowire Layers in the Terahertz Frequency Range

**DOI:** 10.3390/ma14237399

**Published:** 2021-12-02

**Authors:** Aleksandra Przewłoka, Serguei Smirnov, Irina Nefedova, Aleksandra Krajewska, Igor S. Nefedov, Petr S. Demchenko, Dmitry V. Zykov, Valentin S. Chebotarev, Dmytro B. But, Kamil Stelmaszczyk, Maksym Dub, Dariusz Zasada, Alvydas Lisauskas, Joachim Oberhammer, Mikhail K. Khodzitsky, Wojciech Knap, Dmitri Lioubtchenko

**Affiliations:** 1 CENTERA Laboratories, Institute of High Pressure Physics PAS, 01-142 Warsaw, Poland; aleksandra.przewloka@gmail.com (A.P.); aleksandrababis@gmail.com (A.K.); but.dmitry@gmail.com (D.B.B.); kstelmaszczyk@unipress.waw.pl (K.S.); mdub@unipress.waw.pl (M.D.); alvydas.lisauskas@ff.vu.lt (A.L.); knap.wojciech@gmail.com (W.K.); 2 Institute of Optoelectronics, Military University of Technology, 00-908 Warsaw, Poland; 3 Department of Micro and Nanosystems, KTH Royal Institute of Technology, SE-100 44 Stockholm, Sweden; sergueis@kth.se (S.S.); joachim.oberhammer@ee.kth.se (J.O.); 4 Department of Electronics and Nanoengineering, School of Electrical Engineering, Aalto University, 02150 Espoo, Finland; irina.nefedova@aalto.fi; 5 Department of Physics, Saratov National Research State University, 410012 Saratov, Russia; ignefedov@outlook.com; 6 THz Biomedicine Laboratory, ITMO University, 197101 Saint Petersburg, Russia; petr.s.demchenko@gmail.com (P.S.D.); dvzykov@itmo.ru (D.V.Z.); v.chebotarev@gmail.com (V.S.C.); khodzitskiy@yandex.ru (M.K.K.); 7 V. E. Lashkaryov Institute of Semiconductor Physics, National Academy of Sciences of Ukraine, 41 pr. Nauki, 03680 Kyiv, Ukraine; 8 Faculty of Advanced Technologies and Chemistry, Institute of Materials Science and Engineering, Military University of Technology, 00-908 Warsaw, Poland; dariusz.zasada@wat.edu.pl; 9 Institute of Applied Electrodynamics and Telecommunications, Vilnius University, 10257 Vilnius, Lithuania

**Keywords:** silver nanowire, AgNWs, terahertz time-domain spectroscopy, terahertz frequency-domain spectroscopy

## Abstract

Thin layers of silver nanowires are commonly studied for transparent electronics. However, reports of their terahertz (THz) properties are scarce. Here, we present the electrical and optical properties of thin silver nanowire layers with increasing densities at THz frequencies. We demonstrate that the absorbance, transmittance and reflectance of the metal nanowire layers in the frequency range of 0.2 THz to 1.3 THz is non-monotonic and depends on the nanowire dimensions and filling factor. We also present and validate a theoretical approach describing well the experimental results and allowing the fitting of the THz response of the nanowire layers by a Drude–Smith model of conductivity. Our results pave the way toward the application of silver nanowires as a prospective material for transparent and conductive coatings, and printable antennas operating in the terahertz range—significant for future wireless communication devices.

## 1. Introduction

Rapid digitization of the world results in higher and higher expectations for the electronics industry [1]. In particular, the attention is focused on the new group of electronic devices, which are expected to transfer information with speeds going up to hundreds of Gigabits per second. For this reason, high hopes are pinned on terahertz (THz) radiation that can carry high data rates with simplified modulation schemes. The THz frequency range became the subject of intense research only in the 1990s [2,3]. This has been caused by difficulties with designing devices capable of generating, detecting, and modulating the signals [2,4]. The fast development of technology in various fields has opened new doors for broad applications of THz radiation, not only for wireless high data rate communication but also in medical diagnosis, imaging techniques, non-destructive testing, security screening, sensing of contamination in water, food and medicines [3,5,6,7,8,9].

The conventional techniques that are currently used in antenna manufacturing cannot fulfil the growing expectations of certain emerging applications which require antennas with high transparency and flexibility [1]. To address this issue, the present research focuses on implementing functional materials, e.g., graphene [10,11,12], carbon nanotubes [8,13], and metallic nanostructures [14,15,16,17,18,19,20,21], for the design of miniaturized and tunable antennas. The conductivity of the metal defines the antenna performance, such as radiation efficiency [22]. In metal nanowire networks, adjusting the length, diameter, and concentration of wires allows tuning of the optical/THz properties that may lead to improvement of the efficiency of nanowire-based antennas [14,18,23,24]. Metal nanowires, especially silver nanowires (AgNWs), are a prospective material for nanoelectronic circuits, transparent and conductive coatings, printable antennas, and other applications [22,25,26,27,28,29,30]. There are numerous reports on the fabrication of AgNW layers, such as vacuum filtration [31,32], transfer printing [33,34,35], air-spraying from suspension [36,37], and rod-coating technique [38,39]. Nevertheless, all of these methods present several challenges related to the inability to obtain layers that would simultaneously exhibit excellent high-frequency/THz properties, low surface roughness, high transparency, flexibility, and stretchability [35,40]. The direct deposition of AgNW layers by printing seems to be the most attractive and promising technique due to its advantages, such as low cost, the facility of production, and the feasibility of large-scale integration [33,41].

One of the AgNW layers’ essential characteristic (which is crucial for THz antennas and reflective surfaces) is the relatively high and frequency-independent conductivity in a wide frequency range [14,36]. It has been demonstrated that AgNW films with relatively high optical transparency are suitable for plasmonic devices operating in the THz range [25]. Additionally, a hybrid THz slot antenna based on a AgNWs network film was described as a promising device for an extremely sensitive microbial detection [22].

The characterization of nano-sized materials in 2D layers is a challenging problem. The reflection/transmission measurement based on the Nicolson-Ross-Weir parameter extraction algorithm is the most commonly used characterization method [42]. There are also various methods based on cavities and open resonators [43]. These methods, however, are problematic when applied to very thin and lossy materials.

In this article, we present the deposition by vacuum filtration of thin silver nanowire layers of various diameters, lengths, and surface densities. Except for the standard characterization methods (AFM, UV-Vis-IR spectroscopy), we also present systematic transmission and reflectivity measurements in the THz range (0.2 THz to 1.3 THz) with two different systems: pulsed time-domain spectroscopy (TDS) and continuous-wave (CW) frequency-domain spectroscopy. The experimentally observed dependencies are described by establishing the relation between the nanowire layer structure and the network’s electromagnetic response. A modified Drude–Smith model of conductivity indicates that the samples with a low density of nanowires follow the Drude–Smith model with a backscattering coefficient close to −1, and samples with a high density form a semi-continuous metallic layer. Our results indicate that silver nanowires are prospective material for nanoelectronic circuits, transparent and conductive coatings, and printable antennas operating in the terahertz range—significant for 5G and beyond, wireless communications.

## 2. Experimental

### 2.1. Sample Preparation

The samples were prepared from commercial AgNWs in isopropanol (IPA) suspension (MilliporeSigma; Burlington, MA, USA); 5 mg/mL, product numbers 807389, 807176, and 807052) with three nanowire dimensions, detailed in Table 1. The volume equal to 100 µL of the AgNW-IPA suspension was next added to 300 mL of deionized H_2_O and mixed in an ultrasonic bath for 30 min. Uniformly distributed AgNW layers were obtained by vacuum filtration of the diluted suspension with volumes ranging from 2 mL to 30 mL onto polyvinylidene difluoride membranes (MilliporeSigma; HVLP, 0.45 µm pore size, 25 mm diameter, 125 µm thickness). The membranes were chosen as highly transparent in the THz frequency range of interest. This sample preparation method required no additional transfer of the nanowires to different substrates and enabled direct THz characterization of the samples on the membranes. The samples were dried in air and stored in $N_{2}$ atmosphere in the special dry cabinet to avoid degradation of the nanowires, in accordance with the recommendations in the safety data sheet [27].

### 2.2. Sample Characterization

**Figure 1:**
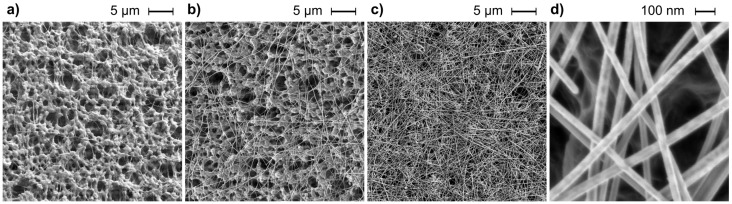
SEM images of AgNWs C samples with (**a**) lowest sample density, showing individual nanowires and the morphology of the porous substrate (dark features), (**b**) intermediate sample density at the percolation threshold, where the nanowires form a connected network, (**c**) highest sample density with the nanowires forming a semi-continuous metallic layer, (**d**) high magnification of (**c**).

**Figure 2:**
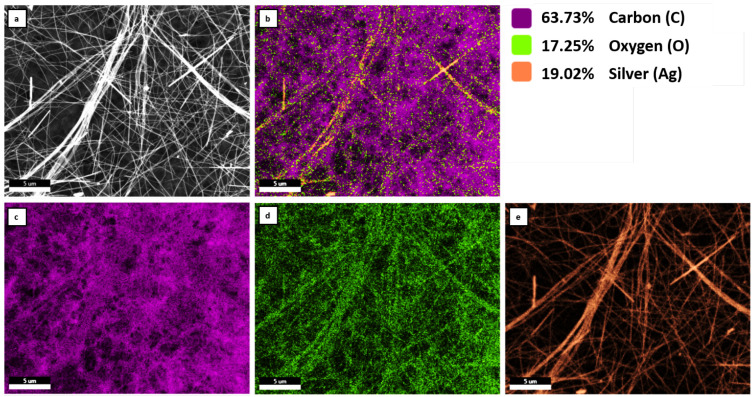
SEM-EDS analysis of AgNWs B sample: (**a**) SEM image of the analyzed area; (**b**) cumulative map of elements distribution in the area of interest with the percentage content of individual elements; distribution maps of carbon (**c**), oxygen (**d**) and silver (**e**).

Scanning electron microscope (SEM) imaging was performed with a high-resolution SEM Zeiss Ultra 55 with a secondary electron detector, an acceleration voltage of 2 kV, and a working distance of 3 mm. Figure 1 shows representative SEM images of the AgNW layers with increasing densities from (a) individual nanowires on the porous substrate, (b) an interconnected nanowire network at approximately the percolation threshold, to (c) a dense nanowire layer forming a continuous network. The AgNWs were distributed on the HVLP membrane uniformly. Please note that the dark circular features in Figure 1a,b originate from the morphology of the porous substrate and the nanowires are represented by the individual bright lines. As can be observed in the Figure 1d at higher magnification, the nanowires surfaces are smooth and free from crystalline oxidation products. Only sample C is illustrated in Figure 1, samples A and B were without any visual differences in the SEM figures. Chemical composition of AgNWs on mixed cellulose esters membrane (MilliporeSigma; MCE, 0.45 µm pore size) was analyzed using a combined scanning electron/focused ion beam Quanta 3D FEG microscopy (SEM/FIB), equipped with the integrated EDAX Pegasus EDS (Energy Dispersive X-Ray Analysis) and EBSD (Electron Backscatter Diffraction) system. Measurements were carried out with the acceleration voltage of 20 kV. Figure 2 exhibits the SEM micrograph of AgNWs on membrane (sample B) with corresponding EDS mapping. The performed EDS analysis confirmed the presence of silver, oxygen and carbon on the sample surface. The percentage weight ratio of Ag, O and C is 19.02, 17.25 and 63.73, respectively (Figure 2b). As can be observed, the presence of carbon is related to the substrate on which the nanowires were deposited (Figure 2c). In the mapping image, oxygen comes also from MCE membrane, but it is also found in the AgNWs region (Figure 2d), which can be explained by the oxidation of AgNWs. The dispersion of Ag on analyzed area is shown in the in Figure 2e. As can be seen, this element is in the area of silver nanowires.

**Figure 3:**
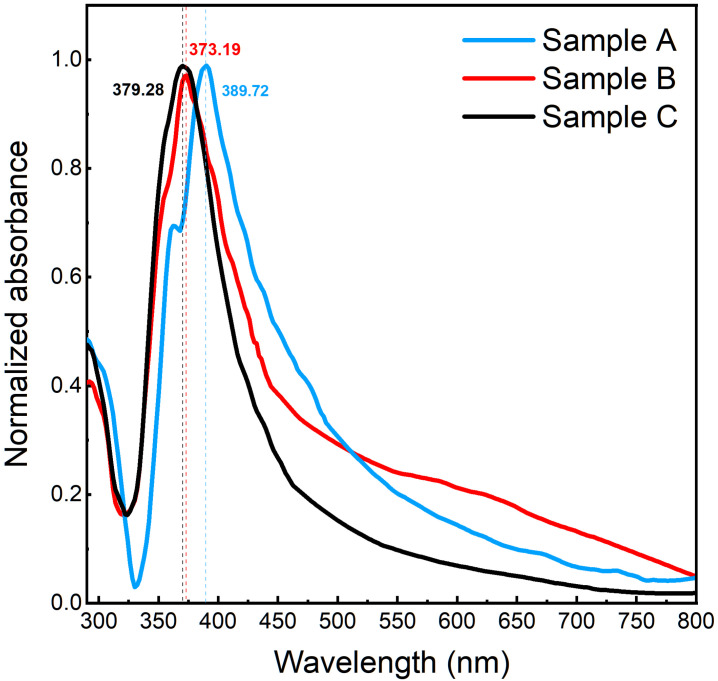
Normalized optical absorbance of the three AgNW sample suspensions.

Atomic force microscopy (AFM) was performed with a Veeco Dimension 5000 system. A typical AFM image of the AgNWs on a PET substrate is shown in Figure S1. From the SEM and AFM images, the samples’ thicknesses were estimated to range from an individual nanowire to a thick, dense layer: approximately between 50 nm and 1 µm. The optical transmittance spectra were obtained with a Perkin Elmer UV-Vis-NIR Lambda 1050+ spectrometer in the wavelength range of 300 nm to 800 nm. The optical absorbance of the samples in solution is shown in Figure 3, with a well-known, characteristic strong peak between 370 nm and 390 nm originating from the transverse plasmon resonance of the nanowires. The optical properties of AgNWs samples depending on the diameter, was previously described in reference [27]. The AgNWs in IPA solution and after the vacuum filtration process were measured with Bruker Vertex 80v Fourier Transform Infrared Spectrometer (FTIR), the spectra are shown in Figure S2.

### 2.3. Terahertz Time-Domain Spectroscopy

A terahertz TDS was used in transmission mode to extract the complex conductance of the AgNWs. An infrared femtosecond laser generates a series of pulses with a 1040 nm central wavelength, 200 fs pulse duration, 70 MHz repetition rate, and 15 nJ pulse energy. The laser beam is split into a probe beam and a pump beam with an energy ratio of 10% to 90%. The path of the pump beam is controlled by an optical delay line and modulated by a chopper at 667 Hz. The THz radiation is generated in an InAs crystal (in a magnetic field of 2 T). After passing through an IR filter, the THz beam incidents on the sample and reaches the CdTe semiconductor detector. The probe beam passes through a half-wave plate, a Glan prism, and meets with the THz beam on the CdTe surface. The polarization of the probe beam varies proportionally to the THz wave amplitude at a given time point, depending on the position of the time delay line. The beam is split into two orthogonally polarized components by a Wollaston prism and detected with balanced photodiodes.

The schematic diagram of the THz-TDS setup is illustrated in Figure S3. A signal to noise ratio of 45 dB is achieved over the frequency range of 0.1 THz to 1 THz. The measurements were carried out at room temperature and relative humidity of 55%. The sample area under study was a circle of a diameter of 5 mm.

### 2.4. Terahertz Frequency-Domain Spectroscopy

A commercial continuous-wave terahertz spectrometer (Toptica TeraScan 1550) was used for the frequency-domain measurements of the samples. The CW-THz spectrometer contains two distributed-feedback diode lasers (laser 1 and laser 2) working in the system using the photomixing technique, where the generated THz signal is equal to the frequency of the laser heterodyne [44]. Scanning of the THz frequency is achieved by cooling one while heating the other laser, which tunes the wavelength around the central value of 1.5 µm. Both lasers are combined to a beating signal via a 50:50 fiber coupler. The beating signal is split into the emitter (Tx) and the receiver (Rx) branch. The beat can be varied continuously from 0 to 1.2 THz, defining the frequency range of the spectrometer, with a practical lower limit of the setup around 50 GHz [45]. The laser beating signal is transformed via a self-complementary broadband antenna on an InGaAs photodiode into the terahertz wave. The photomixers are placed on a hyper-hemispherical silicon lens, which suppresses back-reflections and pre-collimates the THz radiation in free space. The emitter is gated with a DC signal bias and a modulation AC lock-in signal. The receiver is connected to a lock-in signal amplifier. The bare substrate without nanowires and a silicon wafer were measured for reference.

A schematic diagram of the system is illustrated in Figure S4a. The THz quasi-optical feed consists of four parabolic mirrors that can be configured in transmission or reflection with an incident angle of approx 10 degrees. The system achieves a peak dynamic range of 90 dB at 200 GHz (see Figure S4b) and a spectral resolution of 2 GHz. A control silicon wafer and an empty porous membrane were measured for reference (see Figure S5).

## 3. Results and Discussion

Time-domain waveforms of the THz pulses transmitted through the samples (AgNWs on the substrate), the bare substrate, and air as a reference are shown in Figure S6. The amplitude and phase as a function of the frequency were calculated by fast Fourier transform (FFT) of the time-domain pulses. For the CW system, the amplitude spectrum was obtained from the measured frequency-domain photocurrent (see Figure S7). The amplitude of each local maximum was averaged with the adjacent minimum to remove DC offsets in the photocurrent and was linearly interpolated to the original frequency point.

The transmittance for both systems was obtained as the ratio of amplitudes through the samples and the bare substrate (see Figure S8, dashed lines for TDS and solid lines for the CW system). The bare substrate shows a transparency of around 95% in the 0.1 THz to 1.3 THz range due to its highly porous nature. The measurements from both TDS and CW systems prove to be in good agreement showing that both methods are well adapted for characterizing AgNW layers. The samples’ transmittances are decreasing with the increasing nanowire densities and are relatively flat over the measured frequency range. We attribute the differences in transmittance between the samples A, B, and C to differences in the filling factor, i.e., the fraction of nanowires volume to the total volume of the layers. As both the average diameter and length of the nanowires decreases from sample A to C, more nanowires per unit volume are present in samples C than A for the same density.

**Figure 4:**
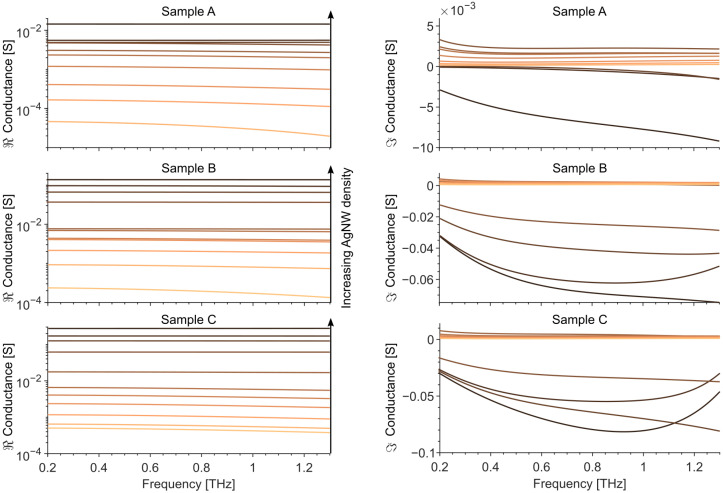
Real and Imaginary parts of the samples’ THz conductance with different nanowire densities, calculated from the TDS measurement data according to (Equation (1)).

The complex conductance of the nanowire layer as a function of the frequency ω was calculated from the measured transmittance as [46]:

(1) $$\hat{\sigma }\left(\omega \right)=\frac{1}{Z_{0}}\left({\hat{n}}_{sub}+1\right)\left(\frac{{\hat{E}}_{0}\left(\omega \right)}{\hat{E}\left(\omega \right)}-1\right),$$

where $Z_{0}$ = 377 Ω is the impedance of free space, ${\hat{n}}_{sub}$ is the complex refractive index of the substrate, and ${\hat{E}}_{0}\left(\omega \right)$ and $\hat{E}\left(\omega \right)$ are the complex electric field amplitudes of THz wave, transmitted through the bare substrate and the substrate with the nanowires. The complex refractive index of the substrate was extracted from a reference measurement of the filter without nanowires. The calculated conductance of the samples is shown in Figure 4.

The measured transmittance was fitted by a physical model of THz conductivity to estimate the 3D filling factor of the nanowires *f* and the layer thickness *h*. The single-scattering approximation of the Drude–Smith model was used in the form [47,48]:

(2) $$\varepsilon_{eff}\left(\omega \right)=\varepsilon_{\infty }-\frac{\omega_{p}^{2}}{\omega (\omega -i\gamma )}\left(1+\frac{C_{1}}{1-i\omega /\gamma }\right),$$

where $\varepsilon_{\infty }=0.11$, $\omega_{p}$ is the effective plasma frequency, γ is the damping factor, and $C_{1}$ is the backscattering parameter. The obtained fitting parameters are given in Table 2 for decreasing nanowire densities of several samples C. The transmittance *T* of the NW layer under normal incidence reads as [27]:

(3) $$T=\frac{2}{2\cos\left(k_{eff}h\right)-i\sin\left(k_{eff}h\right)\left(\sqrt{\varepsilon_{eff}}+1/\sqrt{\varepsilon_{eff}}\right)\prime }$$

where $k_{eff}=k\sqrt{\varepsilon_{eff}}$, and *k* is the wavenumber in free space. The reflection coefficient is expressed as:

(4) $$R=1-T\left[\cos\left(k_{eff}h\right)-i\sqrt{1/\varepsilon_{eff}}\sin\left(k_{eff}h\right)\right].$$

**Figure 5:**
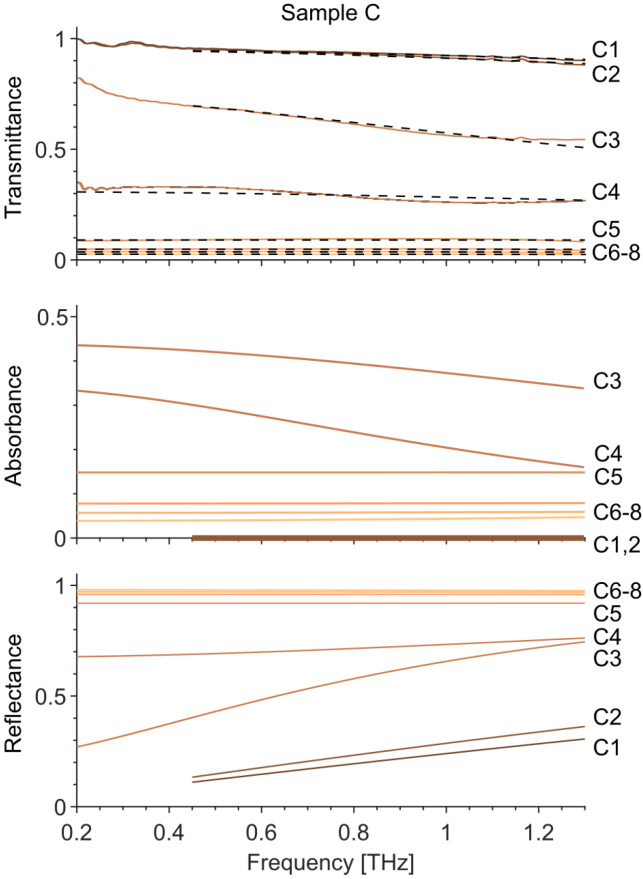
Transmittance (solid lines—measured, dashed lines—calculated), Absorbance and Reflectance (calculated) of AgNWs samples C with different nanowire densities extracted from the fitted Drude–Smith conductivity model according to (Equations (3) and (4)) and Table 2.

The absorbance and reflectance were calculated according to $A=1-{\left|T\right|}^{2}-{\left|R\right|}^{2}$. The calculated and fitted *T*, *A*, and *R* are shown in Figure 5 for AgNW samples C with different densities.

The fitted layer thicknesses *h* match those observed with AFM and SEM. For the samples with the lowest nanowire densities (C1 to C4), the effective plasma frequency is taken equal to the one of bulk silver $\omega_{p,Ag}=1.32\cdot 10^{16}$ rad/s. The fitted scattering parameter $C_{1}$ is close to −1, which indicates high carrier localization in the nanowires and preferential backscattering. Such behavior can be explained by the backscattering of electrons from the nanowire walls, usually observed for networks below the percolation threshold. For the samples with the highest nanowire densities (C5 to C8), the scattering parameter $C_{1}$ is set to 0, equal to Drude-like scattering. In this case, the conductivity corresponds to a semi-continuous metallic layer, with an effective plasma frequency taken as $\omega_{p}^{2}=\omega_{p,Ag}^{2}\cdot f$, with *f* the filling factor of the nanowire network. These two THz conductivity models explain the measured effective conductivity, where the imaginary part is negative for high density samples (C5 to C8) and positive for low density samples (C1 to C4). We would like to stress that the theoretical approach describes well the experimental results and therefore it shows that can be used for modelling/predicting the THz response as a function of the nanowire layer structure.

The fitted parameters are compared to those found in the literature for bulk silver and AgNW layers fabricated by different deposition methods in Table 3. Significant differences are observed in the range of THz conductivities and carrier scattering times that we mainly attribute to the differences in cross-section area of the nanowires. The comparative table highlights the importance of the selection of nanowire dimensions for achieving the desired THz performance of thin AgNW layers.

## 4. Conclusions

We have deposited and experimentally characterized thin layers of silver nanowires in the 0.2 THz to 1.3 THz frequency range. In particular, samples with three different nanowire morphologies and increasing densities were measured in a transmission geometry by terahertz spectroscopy, both in time and frequency domains. The results obtained from the two systems are in good agreement and allow the validation of both experimental methods for the characterization of silver nanowire layers. We extracted from the measurements the complex conductance of the samples with varying densities, which exhibits a real part that ranges over two orders of magnitude (roughly between 1 mS and 100 mS) and an imaginary part that shows a transition from negative to positive values. The results were fitted with a modified Drude–Smith model of conductivity. The samples with a low density of disconnected nanowires follow the Drude–Smith model with a backscattering coefficient close to −1, indicating high localization of electrons in the nanowires. The samples with a high density form a semi-continuous metallic layer that follows a Drude-like model of conductivity with an effective plasma frequency adjusted by the 3D filling factor of the nanowires. The relatively constant conductance of the nanowire layers in a broad frequency range is of particular interest, as tunable transparent coatings are distinctly demanded for high-frequency applications. Knowledge of the THz permittivity and conductivity of silver nanowire networks paves the way toward the application of silver nanowires as a prospective material for nanoelectronic circuits, transparent and conductive coatings, and printable THz antennas, essential for future wireless communication systems.

## Figures and Tables

**Table 1:** Nanowire dimensions.

Sample	Diameter (nm)	Length (µm)
A	40±5	35±5
B	35±5	25±5
C	30±5	20±3

**Table 2:** Drude–Smith fitting parameters for AgNW samples C.

Sample	*h* (nm)	*f*	$C_{1}$	γ (Hz)
C8	1200	0.35	0	$2.45\cdot 10^{15}$
C7	800	0.2	0	$1.4\cdot 10^{15}$
C6	570	0.1	0	$7.0\cdot 10^{14}$
C5	275	0.08	0	$5.6\cdot 10^{14}$
C4	250	0.085	−0.983	$5.95\cdot 10^{14}$
C3	180	0.08	−0.997	$5.6\cdot 10^{14}$
C2	80	0.1	−1	$7.35\cdot 10^{14}$
C1	80	0.11	−1	$7.7\cdot 10^{14}$

**Table 3:** Comparison of the THz properties of AgNWs found in the literature. *d*—nanowire diameter, *l*—nanowire length, *h*—layer thickness, *f*—filling factor, ω—frequency range, σ—real part of the THz conductivity, $C_{1}$—backscattering parameter, $\omega_{p}$—effective plasma frequency, 1/γ—carrier scattering time. N.A.—not applicable, “—” indicates that data have not been reported. Adapted from [36], with the permission of AIP Publishing. Adapted with permission from [49], © 2014 American Chemical Society.

	Nanowire	*d*	*l*	*h*	*f*	ω	σ	$C_{1}$	$\omega_{p}/2\pi$	1/γ
Ref.	Deposition	(nm)	(µm)	(nm)	(%)	(THz)	(S/cm)		(THz)	(fs)
[50]	Bulk Ag	N.A.	N.A.	N.A.	100	5.4–600	$6\cdot 10^{5}$	0	2181	230
[36]	Bar coating	90±10	5±2	Monolayer	1.5–14.8	0.3–1.5	0–50	−0.99	174–187	25–28
[49]	Spin coating	70–100	10	—	8–30	0.4–2	300–1600	−0.9–0	300–1500	20–80
[30]	Spray coating	50	10	120–240	—	0.2–2	4–830	—	—	—
Here	Vacuum filter.	30±5	20±3	80–1200	8–35	0.2–1.3	4–230	−1–0	590–2100	0.4–1.8

## Data Availability

Publicly available datasets were analyzed in this study. This main data can be found here: http://urn.kb.se/resolve?urn=urn:nbn:se:kth:diva-305516 (accessed on 27 September 2021) Additional data are available on request from the corresponding author.

## Supplementary Materials

The following are available online at https://www.mdpi.com/article/10.3390/ma14237399/s1, Figure S1: AFM image of the AgNWs network, Figure S2: FTIR spectrum of AgNWs, Figure S3: Schematic diagram of the THz-TDS system, Figure S4: (a) Schematic diagram of the THz-CW spectrometer. (b) The signal-to-noise ratio of the system, Figure S5: Measured transmittance of a bare substrate and a silicon wafer as reference, Figure S6: Measured time-domain THz pulses through the air as a reference, the substrate, and several samples A with different nanowire densities, Figure S7: Measured frequency-domain THz photocurrent through the air as a reference, the substrate, and several samples A with different nanowire densities, Figure S8: Measured amplitude transmittance through the samples (normalized to the substrate) with increasing nanowire densities.
