# Supplementary material for: Characterization of Silver Nanowire Layers in the Terahertz Frequency Range
Source: Materials (Basel). 2021 Dec 2;14(23):7399. doi: 10.3390/ma14237399 (PMC8658758; doi:10.3390/ma14237399)
Supplement: Supplementary file 1 [file materials-14-07399-s001.zip › materials-1419967-supplementary.pdf]

Supplementary Materials: Characterization of silver nanowire layers in the terahertz frequency range

Aleksandra Przewłoka [1,2,†], Serguei Smirnov [3,†], Irina Nefedova [4], Aleksandra Krajewska [1], Igor S. Nefedov [5], Petr S. Demchenko [6], Dmitry V. Zykov [6], Valentin S. Chebotarev [6], Dmytro B. But [1], Kamil Stelmaszczyk [1], Maksym Dub [1,7], Dariusz Zasada [8], Alvydas Lisauskas [1,9], Joachim Oberhammer [3], Mikhail K. Khodzitsky [6], Wojciech Knap [1], and Dmitri Lioubtchenko [1,3*]

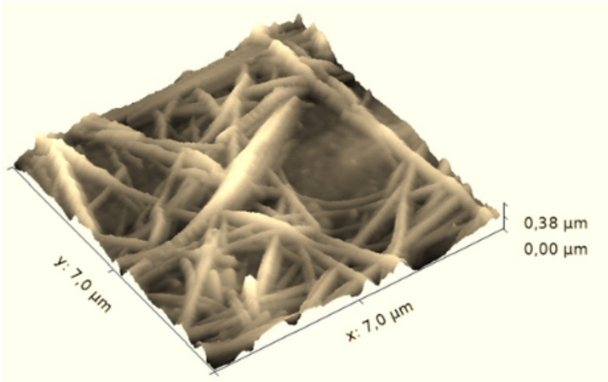

Figure S1. AFM image of the AgNWs network.

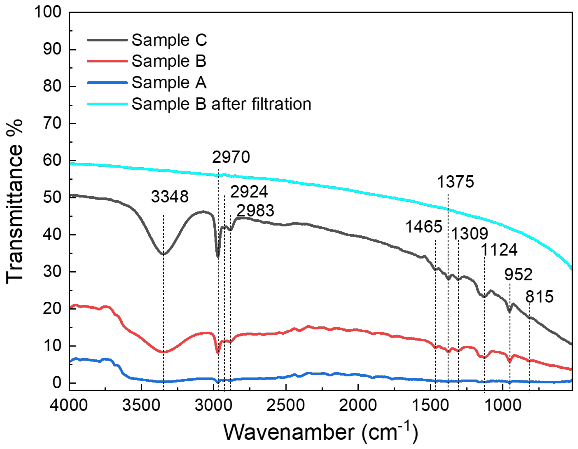

Figure S2. FTIR spectrum of AgNWs.

The transmittance spectra of AgNWs in isopropanol solution and spectrum AgNWs B sample after the vacuum filtration process were measured with Bruker Vertex 80v Fourier Transform Infrared Spectrometer (FTIR). The representative FTIR spectra are shown in Fig. S2. The presence of the peaks at the wavenumber of 3348 cm^{-1} (OH groups of solvents), 2970 cm^{-1} (CH), 2924 cm^{-1} is assigned to asymmetric stretching vibration of CH_2. The peak 1465 cm^{-1} is corresponded to CH_2 stretching and bending. The peak 1375 cm^{-1} can be assigned to the stretching vibrations of C=H. Compared to the spectrum of the AgNWs B sample after vacuum filtration, peaks at the above-mentioned values are not observed. This indicates that the deionized water and IPA were completely removed by vacuum filtration. The transmission of AgNWs samples depending on the diameter, was previously described in reference [27].

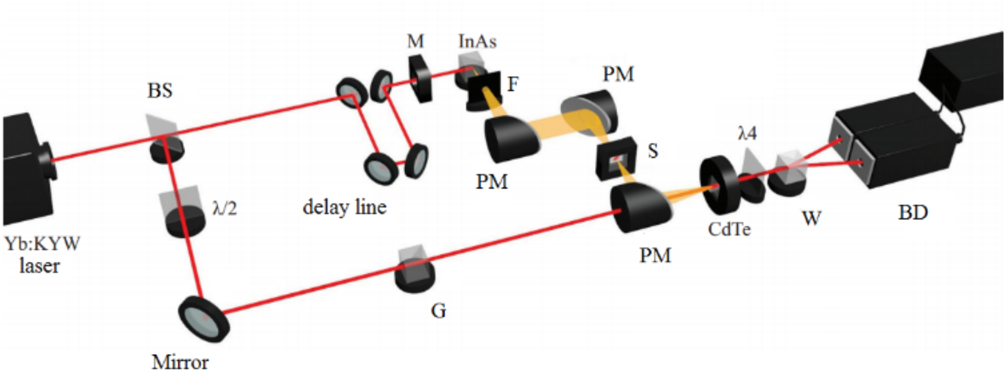

Figure S3. Schematic diagram of the THz-TDS system. BS – beam splitter, M – modulator, F – IR filter, S – sample, PM – parabolic mirror, G – Glan prism, W – Wollaston prism, BD – balanced detector.

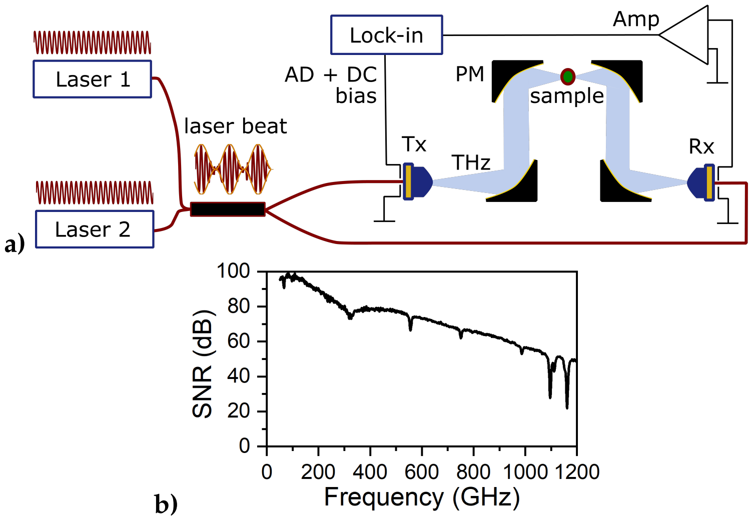

Figure S4. a) Schematic diagram of the THz-CW spectrometer. Tx – emitter, Rx – receiver, PM – parabolic mirror. b) The signal-to-noise ratio of the system.

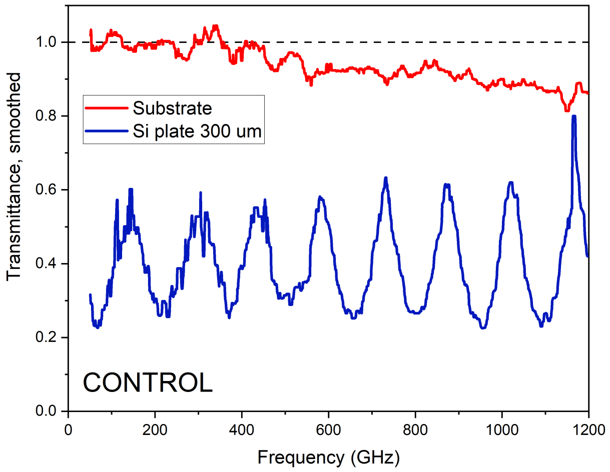

Figure S5. Measured transmittance of a bare substrate and a silicon wafer as reference.

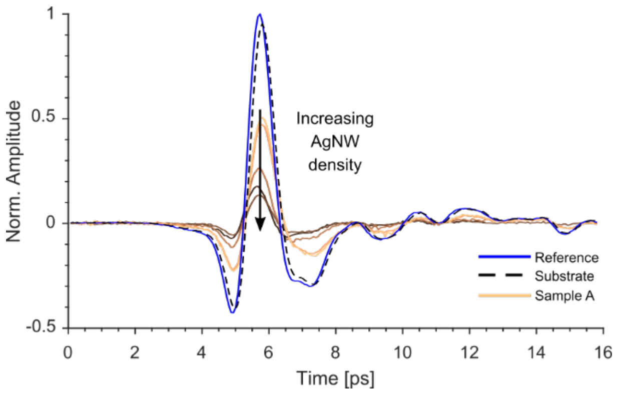

Figure S6. Measured time-domain THz pulses through the air as a reference, the substrate, and several samples A with different nanowire densities.

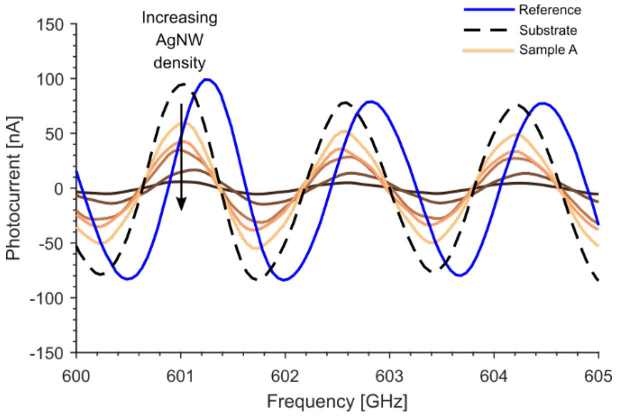

Figure S7. Measured frequency-domain THz photocurrent (zoomed to a small frequency range for better visibility) through the air as a reference, the substrate, and several samples A with different nanowire densities. The spacing between maxima indicates a spectral resolution of 2 GHz.

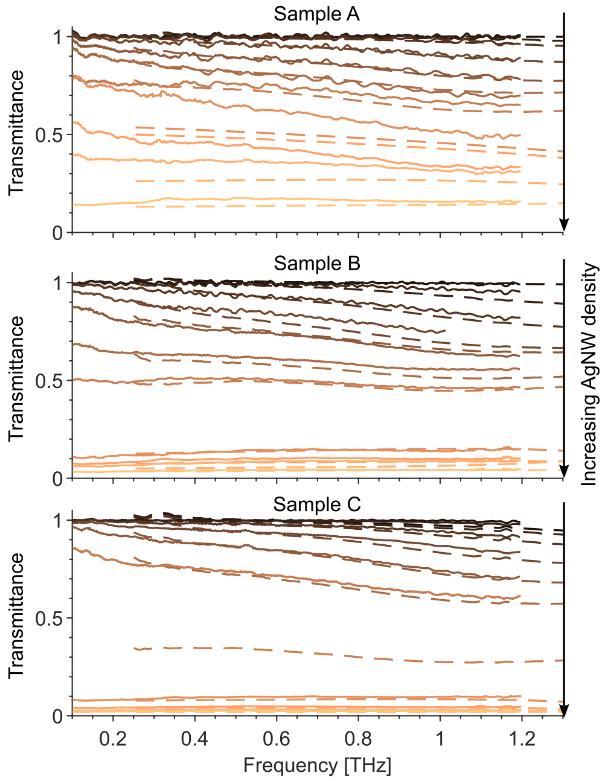

Figure S8. Measured amplitude transmittance through the samples (normalized to the substrate) with increasing nanowire densities – dashed lines with the TDS and solid lines with the CW system.
